# Current News

**Published:** 1895-03

**Authors:** 


### Current News.

### ANNUAL MEETING OF THE INTERNATIONAL DENTAL PUBLICATION COMPANY.

The annual meeting of the International Dental Publication Company took place at Jersey City, on January 26, at which the report of the board of directors was received and the election of officers was held.

From the report it appears that the financial interests of the journal have not suffered from the general business depression as had been anticipated, the residue of income having been considerably greater than for the previous year.

It was expressed that the profession has not met the laudable and energetic efforts made to supply the profession with an independent journal with the amount of support to which the board believed the INTERNATIONAL DENTAL JOURNAL, from its merits, to be entitled. They state it has become evident that the conduct of the JOURNAL in a business direction must become more active. In agreement with this impulse an appeal is being made to the leading dental societies to take some action towards influencing subscriptions for the JOURNAL on the ground that it belongs to the profession, and that it should for this reason be loyally and amply sustained.

We quote from the editor's report,—

"The result of the year's work is certainly in many respects remarkable. From the best authorities it is learned that the past year, 1894, was the most trying year periodicals of all kinds have experienced, and a journal that could hold its own and show no deficit was regarded as doing very well. As our journal has done more than this, it may be regarded as exceptionally successful, and should be held as firmly established.

"The present period of depression will necessarily be slowly dissipated, but following the general law of all crises in the financial world, it cannot well last over another year, and possibly the present may show a marked improvement. It must be regarded, therefore, as certain that we have passed the most difficult period, and with persistent economy we ought to reach finally the open sea of successful journalism.

"It is regarded now as the only exponent of independent dental thought conducted upon a high standard of matter and character of production. It is possible that some may have been disappointed, that it has not waged war on trade influences. In a quiet way the JOURNAL has been a serious obstruction to the complete success of certain trade journals. This has been made evident in many directions. It is thought that, for the present, the best line of antagonism to these journals lies in thorough work and a quiet influence with societies to rise to the standard of higher professional spirit.

"The evil influence of trade has so permeated the entire body that any mere series of editorial denunciations will not avail. The body must be educated to a higher life, and this will take time and persistent effort.

"It is with this view that the editor has refrained from any direct attack on trade organizations for their manifest interference with the best interest of the profession. It is, however, his opinion that we are fast approaching a time when it will be necessary to strike more decided blows in this direction.

"We cannot close this report without expressing our most grateful thanks to those of the stockholders who have earnestly and persistently stood by the JOURNAL. To the societies who have continued to give it their proceedings, in the face of continual financial offers from capital, the JOURNAL company should express in proper terms its appreciation. The *editor* appreciates this, perhaps, more keenly than others, as he is constantly brought in contact with the under current of competition from capital that, but for the brave opposition of some societies, would be discouraging. So far there has been no break in the band of faithful colaborers,—the New York Odontological Society, Academy of Dental Science, Harvard Odontological Society, Odontological Society of Pennsylvania, and the recently added Academy of Stomatology. The Central Dental Association of Northern New Jersey has contributed largely but not regularly."

The following officers were elected for the ensuing year:

President, Louis Jack; Vice-President, Benjamin Lord; Secretary, George S. Allan; Treasurer, C. N. Peirce.

*Advisory Board.*—E. T. Darby, D. N. McQuillen, S. G. Perry, W. W. Walker, J. A. Woodward, *Chairman.*

*Board of Directors.*—George S. Allan, New York; R. R. Andrews, Cambridge; J. N. Crouse, Chicago; Edwin T. Darby, Philadelphia; J. Morgan Howe, New York; Louis Jack, Philadelphia; V. H. Jackson, New York; H. J. McKellops, St. Louis; D. N. McQuillen, Philadelphia; C. N. Peirce, Philadelphia; S. G. Perry, New York; W. H. Potter, Boston; W. W. Walker, New York; C. A. Woodward, New York; J. A. Woodward, Philadelphia.

---

### THE HORACE WELLS PERMANENT MEMORIAL, UNDER THE AUSPICES OF THE AMERICAN DENTAL ASSOCIATION.

TO THE DENTAL PROFESSION OF AMERICA:

THE Central Executive Committee, appointed by the president of the American Dental Association, is as follows: Dr. James Truman, Dr. Wilbur F. Litch, Dr. S. H. Guilford, Dr. E. C. Kirk, Dr. J. D. Thomas, chairman and treasurer.

This committee has been completed in its organization by including in its membership the presidents of all dental societies throughout the United States.

It is hoped to secure enough money to erect a bronze statue of Horace Wells in the national capital. The details as to style and character of the statue, as well as its definite location, will be decided upon at the next meeting of the American Dental Association, to be held at Asbury Park, N. J.

The committee takes pleasure in calling your attention to this opportunity for doing an act of justice to the memory of a worthy member of our profession, whose discovery has been of such incalculable benefit to humanity, and which has been so great an honor to our profession. You are invited to contribute whatever amount of money you may feel able and willing to donate to the fund, and to use your influence towards bringing our plan to a successful issue in a manner befitting the object.

Contributions may be sent by any member of the profession through the president of his local society, or direct to the treasurer, Dr. J. D. Thomas, 912 Walnut Street, Philadelphia. An official receipt will be issued by the treasurer for all contributions. The full list of contributors will be embodied in the pedestal of the memorial.

J. D. Thomas,
*Chairman of the Central Executive Committee.*

The Executive Committee requests that all who desire copies of the souvenir volume of the meeting held at Philadelphia, December 11, 1894, in celebration of the fiftieth anniversary of the discovery of anæsthesia by Horace Wells, will promptly forward their names to the chairman, in order that the number of copies to be printed may be determined upon. The price has been fixed at $1.50 per volume, postage free to all parts of the United States; foreign countries at regular postage rates.

---

### FIRST DISTRICT DENTAL SOCIETY OF NEW YORK.

An all-day clinic will be held under the auspices of the First District Dental Society of New York, on Tuesday, March 12, at the New York College of Dentistry, 205 East Twenty-third Street, New York City.

A cordial invitation is extended to all members of the profession to be present. Any one desiring to clinic, or exhibit anything of value, is requested to communicate with the committee.

James G. Palmer,
*Chairman Clinic Committee.*

18 West Thirty-fifth Street, New York City.

